# When Lack of Trust in the Government and in Scientists Reinforces Social Inequalities in Vaccination Against COVID-19

**DOI:** 10.3389/fpubh.2022.908152

**Published:** 2022-07-20

**Authors:** Nathalie Bajos, Alexis Spire, Léna Silberzan, Antoine Sireyjol, Florence Jusot, Laurence Meyer, Jeanna-Eve Franck, Josiane Warszawski, The EpiCov study group, Nathalie Bajos

**Affiliations:** ^1^Institut de Recherche Interdisciplinaire sur les Enjeux Sociaux (IRIS), Institut National de la Santé et de la Recherche Médicale (INSERM)/École des Hautes Études en Sciences Sociales (EHESS), Aubervilliers, France; ^2^Institut de Recherche Interdisciplinaire sur les Enjeux Sociaux (IRIS), Centre National de la Recherche Scientifique (CNRS), Aubervilliers, France; ^3^Institut de Recherche Interdisciplinaire sur les Enjeux Sociaux (IRIS), Institut National de la Santé et de la Recherche Médicale (INSERM), Aubervilliers, France; ^4^Laboratoire d'Économie et Gestion des Organisations de Santé, Université Paris Dauphine, Paris, France; ^5^Centre de Recherche en Epidémiologie et Santé des Population, Institut National de la Santé et de la Recherche Médicale, Université Paris-Saclay, Le Kremlin-Bicêtre, France

**Keywords:** vaccination, trust, government, scientists, COVID-19, social inequalities

## Abstract

**Objective:**

To assess whether lack of trust in the government and scientists reinforces social and racial inequalities in vaccination practices.

**Design:**

A follow-up of the EpiCov random population-based cohort survey.

**Setting:**

In July 2021, in France.

**Participants:**

Eighty-thousand nine hundred and seventy-one participants aged 18 years and more.

**Main Outcome Measures:**

Adjusted odds ratios of COVID-19 vaccination status (received at least one dose/ intends to get vaccinated/ does not know whether to get vaccinated/refuses vaccination) were assessed using multinomial regressions to test associations with social and trust factors and to study how these two factors interacted with each other.

**Results:**

In all, 72.2% were vaccinated at the time of the survey. The population of unvaccinated people was younger, less educated, had lower incomes, and more often belonged to racially minoritized groups, as compared to vaccinated people. Lack of trust in the government and scientists to curb the spread of the epidemic were the factors most associated with refusing to be vaccinated: OR = 8.86 (7.13 to 11.00) for the government and OR = 9.07 (7.71 to 10.07) for scientists, compared to vaccinated people. Lack of trust was more prevalent among the poorest which consequently reinforced social inequalities in vaccination. The poorest 10% who did not trust the government reached an OR of 16.2 (11.9 to 22.0) for refusing to be vaccinated compared to the richest 10% who did.

**Conclusion:**

There is a need to develop depoliticised outreach programmes targeted at the most socially disadvantaged groups, and to design vaccination strategies conceived with people from different social and racial backgrounds to enable them to make fully informed choices.

## Introduction

Clinical trials have shown the efficacy of the COVID-19 vaccines on SARS-CoV-2 infections (1). However, vaccination is efficient in combating the spread of the epidemic, as well as in reducing social inequalities in morbidity and mortality, provided that it is affordable and accessible (2, 3). Still, making COVID-19 vaccines available does not necessarily lead to a very large population vaccine coverage, as shown by the percentages of people who are still not vaccinated in Western countries (2), even when these vaccines are free. Recent studies in the UK, in the US and in Norway (4–8) have shown that the most socially disadvantaged and racially minoritized groups are the least vaccinated. In light of their high risk of infection and mortality from COVID-19 (1), it appears all the more important to understand why they are less likely to be reached by COVID-19 vaccination programmes.

Social barriers hampering access to preventive practices, such as social distance from health professionals, geographical distance from health centers, or experiences of discrimination in the health system (9, 10) need to be taken into account to study this particular preventive practice that is vaccination. Preventive health behaviors can also be influenced by institutional trust, which refers to citizens' beliefs that institutions act transparently and fairly, in accordance with the public interest (11). In a context where governments and scientists have taken the lead in managing the pandemic crisis, it is all the more important to analyse vaccination practices along with consideration of the trust that people place in the government (12–15). Many studies have shown that COVID-19 vaccination intentions are related to trust in the government (11, 15–20). One study, in the UK, simultaneously considered trust in the government and in scientists: COVID-19 hesitancy was associated with low trust in scientists and doctors but the correlation was weaker with trust in government (16). Furthermore, conspiracy beliefs and social media use can also predict vaccine hesitancy (21, 22). Three types of mechanisms can explain the link between institutional trust and vaccination. At a first level, people who trust institutions such as the government and science, are more likely to believe in the messages these actors promote in favor of vaccination (23). Secondly, people who lack trust in the government may consider the refusal of vaccination as a political act of resistance (17). Thirdly, people who distrust the government and scientists are more likely to believe information sources that present vaccines as unnecessary or dangerous (24).

Beyond vaccination intentions, there is now a need to clarify whether, and to what extent, lack of trust in institutions, and particularly in the government, has impacted vaccination practices. Because underprivileged social groups are known to be particularly distrustful of the government (25–27), it could be assumed that the government's strong involvement in vaccination programmes and its resulting high degree of politicization are likely to reinforce social inequalities in vaccination. In France as in many countries, the government strongly relied on scientists to justify its epidemic response actions. Studying the impact of trust in the government on vaccination practices therefore also implies taking trust in scientists into account. Vaccination was made available in France, as of mid-January 2021 for people over 75. People over 50 with a COVID-19-related-comorbidity could get vaccinated as of mid-February. The vaccination campaign was then extended to include all individuals over 55 as of mid-April 2021, and any individual aged 18 and over, as of May 12th 2021. The survey took place in July 2021, i.e., at a time when COVID-19 vaccines were free and readily available in France for any individual aged 18 and over. It was just before anti-COVID certifications became compulsory to access certain public spaces and services. Nonetheless, these certifications were then not too restrictive, as they could be obtained with a full vaccination scheme or with a 72-h-negative-test (PCR or antigenic), which were still free and readily available in France at that time. The objectives of this article were (i) to identify social differences in vaccination status and trust in the government and scientists, and (ii) to investigate whether the lack of trust in the government and scientists increased social inequalities in vaccination practices.

## Participants and Methods

Individuals aged 15 years or older living in France were randomly selected from the FIDELI administrative sampling framework to participate to the EpiCov survey, covering 96.4% of the population, providing postal addresses for all, and e-mail addresses or telephone numbers for 83.0% of them. Differential sampling was used to ensure oversampling of the less densely populated *départements* (i.e., French Administrative Districts), and lower-income categories. Residents in retirement homes were excluded. All selected individuals were contacted by mail, e-mail and text messages, with up to seven reminders. Computer-assisted-web interviews (CAWI) or computer-assisted-telephone interviews (CATI) were offered to a random 20% subsample. The remaining 80% were assigned to CAWI exclusively. All first- and second-round respondents were eligible for the third in July 2021 (28). In this third wave, 85,032 participated in the third wave (79.0% from the second wave and 63.3% from the first one) and served as the basis for this analysis. We focused on people living in metropolitan France and aged 18 and over since vaccination was allowed only for adults at the time of the survey. In all, 80,971 (95.2%) individuals were included in our study.

The survey was approved by the CNIL (French independent administrative authority responsible for data protection) on April 25th 2020 (ref: MLD/MFI/AR205138) and by the “Comité de protection des personnes” (French equivalent of the Research Ethics Committee) on April 24th. The survey also obtained an agreement from the “Comité du Label de la statistique publique,” proving its consistency with statistical quality standards.

### Outcome Measures

Vaccination status was classified into four categories: vaccinated (at least one dose); intends to be vaccinated; does not know whether to get vaccinated; refuses vaccination.

Vaccinated people were also asked to give the date of their first injection.

### Socio-Demographic Variables

We considered the following variables: age, gender, ethno-racial status (based on migration history), having children, social class (based on current or most recent occupation), if the respondent was a healthcare professional, standard of living (based on decile of income per household consumption unit) and formal education (defined according to the French hierarchical grid of educational qualifications), the household and the population size of the municipality. The ethno-racial status, used for the first time in France in a COVID survey, distinguished the mainstream population, i.e., people residing in metropolitan France who are neither immigrants nor native to French Overseas Departments (FOD, i.e., Martinique, Guadeloupe, Reunion Island, Guyane and Mayotte), nor descendants of immigrant(s) or native to FOD. For the minority population, a distinction was made between first-generation (immigrants) and second-generation (descendants of immigrants) immigrants, and the country of origin. The term racially minoritized groups refers to immigrants or descendants of immigrants from the Maghreb, Turkey, Asia and sub-saharan African countries (29).

### Health Variables

Health variables included the existence of COVID-19 comorbidities (i.e., asthma or other respiratory diseases, high blood pressure or cardiovascular diseases, diabetes, cancer, HIV, mental or psychiatric disability, or BMI>30 kg/m^2^) and if the respondent had had a positive COVID-19 test in the past 6 months.

### Trust Variables

Specific interest was finally devoted to the level of trust in the government (“To curb the spread of the coronavirus, what is the level of trust you place in the actions undertaken by (i) the government and (ii) by scientists?”: Complete trust/Fair amount of trust/Little trust/No trust at all/You do not know).

### Statistical Analyses

A first univariate analysis was performed, to compare the distribution of the four vaccination status categories according to social characteristics and trust variables. Then, the cumulative monthly rates of vaccination (from January 31st to June 30th 2021) were stratified by vaccination age categories (18–54/55–74/75+), and assessed according to formal education, standard of living and ethno-racial status.

A multinomial regression was developed to compare the vaccinated people to the others (intend to be vaccinated; do not know whether to get vaccinated; refuse vaccination) and to investigate how non-vaccinated people differed among themselves according to social and trust variables.

We created variables divided into 12 categories crossing a binary variable characterizing the trust variable (Complete trust/Fair amount of trust vs. Little trust/No trust at all/You do not know, labeled as Trust+/Trust-) and formal education or standard of living or ethno-racial status. Six multinomial regressions were then performed, each one adjusted for one combining variable at a time.

Final calibrated weights were calculated to correct for non-response, as detailed elsewhere (28) for the first, second and third waves of the EpiCov survey. Response homogeneity groups were derived from the sampling weight divided by the probability of response estimated with logit models adjusted for auxiliary variables potentially linked to both the response mechanism and the main variables of interest in the EpiCov survey (age, gender, educational level, and region). The percentages presented are weighted to account for the sampling design with unequal inclusion probabilities due to an oversampling of low-income populations and correction of nonresponse bias.

A *P*-value <0.05 was considered statistically significant for multivariate analyses. Given the sample size, the observed differences were consistently statistically significant. Therefore, no tests are presented for univariable analyses.

Individuals who answered that they did not wish to respond to the question on their vaccination status and/or the date of their first injection were excluded (n = 1,93 0.2%). Missing data was rare for all variables (<4%) and was deleted in multivariate analyses (n = 7,068 8.7% excluded).

## Results

The distribution of the vaccination status in the population is presented in Table 1. In all, 72.2% were vaccinated, with at least one injection in July 2021 (71.1% for men vs. 73.3% for women). Less than one respondent out of ten (8.1%) refused to get vaccinated (8.2% of men and 8.1% of women), while one in ten (9.0%) said they intended to get vaccinated (10.7% for men and 9% for women), and a similar proportion did not yet know whether or not they would get vaccinated (10.0% for men and 9.7% for women).

**Table 1:** Distribution of the vaccinal status of people aged 18 years or over living in metropolitan France, by socio-demographic characteristics and trust variables.

	**Had at least one dose**	**Intends to get vaccinated**	**Does not know yet**	**Refuses to get vaccinated**
**Total**	72.2 (62,418)	9.8 (6,746)	9.8 (6,383)	8.1 (5,231)
**Sex**				
Men	71.1 (27,879)	10.7 (3,104)	10.0 (2,625)	8.2 (2,116)
Women	73.3 (34,539)	9.0 (3,642)	9.7 (3,758)	8.1 (3,115)
**Age**				
18–24	54.7 (4,456)	17.2 (1,204)	16.0 (953)	12.1 (712)
25–34	54.1 (5,431)	15.7 (1,281)	15.5 (1,217)	14.7 (1,097)
35–44	60.5 (9,022)	13.6 (1,514)	14.7 (1,526)	11.3 (1,229)
45–54	70.8 (12,360)	10.6 (1,403)	10.5 (1,313)	8.1 (1,064)
55–64	81.7 (13,679)	6.4 (850)	6.5 (888)	5.5 (705)
65–74	89.8 (12,170)	3.8 (404)	3.7 (378)	2.8 (308)
75–84	93.2 (4,449)	2.2 (67)	2.4 (87)	2.2 (80)
85+	88.2 (851)	3.2 (23)	3.3 (21)	5.3 (36)
**Social class**				
Manual workers	64.8 (6,103)	11.7 (951)	12.8 (988)	10.7 (811)
Self-employed and entrepreneurs	75.9 (3,274)	8.2 (304)	7.8 (281)	8.1 (291)
Senior executive professionals	83.1 (17,783)	6.9 (1,250)	5.6 (968)	4.4 (726)
Middle executive professionals	74.8 (17,322)	8.9 (1,679)	8.6 (1,624)	7.6 (1,363)
Employees	70.6 (13,963)	10.0 (1,676)	10.8 (1,795)	8.6 (1,516)
Students	53.8 (1,891)	19.4 (588)	16.0 (414)	10.9 (268)
Never worked	65.7 (601)	11.0 (94)	12.3 (104)	11.0 (74)
Farmers	77.3 (873)	9.8 (104)	7.2 (85)	5.8 (80)
Missing	608	100	124	102
**Formal education**				
No diploma	69.8 (2,795)	10.1 (350)	10.9 (405)	9.2 (314)
Primary education	78.5 (5,209)	7.8 (533)	7.8 (479)	5.8 (353)
Vocational secondary	71.8 (11,936)	9.4 (1,178)	9.7 (1,231)	9.0 (1,062)
High school	66.0 (12,140)	12.1 (1,715)	11.6 (1,556)	10.3 (1,409)
High school +2 to 4 years	72.2 (18,679)	9.7 (1,931)	10.4 (1,924)	7.7 (1,510)
High school +5 or more years	79.2 (11,659)	8.8 (1,039)	6.9 (788)	5.0 (583)
**Standard of living (in deciles)**				
D1	54.8 (3,339)	14.5 (705)	16.9 (745)	13.8 (629)
D2–D3	62.7 (5,939)	12.5 (1,096)	13.6 (1,141)	11.3 (949)
D4–D5	69.9 (8,560)	10.9 (1,229)	10.8 (1,199)	8.4 (987)
D6–D7	75.3 (12,855)	8.5 (1,356)	8.5 (1,305)	7.6 (1,165)
D8–D9	81.9 (18,907)	6.8 (1,474)	6.5 (1,358)	4.9 (974)
D10	87.6 (11,506)	5.6 (641)	3.8 (428)	3.0 (332)
Missing	1,312	245	207	195
**Ethno-racial status**				
Mainstream population	74.5 (52,430)	9.1 (5,285)	8.6 (4,890)	7.8 (4,203)
Born or parents born in FOD	56.2 (554)	14.0 (112)	15.5 (125)	14.2 (96)
Non-racially minoritized second-generation immigrants	75.6 (3,439)	8.7 (350)	8.5 (327)	7.1 (257)
Racially minoritized second-generation immigrants	52.5 (1,490)	15.5 (342)	19.3 (409)	12.8 (251)
Non-racially minoritized first-generation immigrants	76.3 (2,006)	8.5 (164)	8.2 (138)	7.0 (134)
Racially minoritized first-generation immigrants	59.1 (1,654)	16.4 (377)	17.2 (354)	7.4 (170)
Missing	845	116	140	120
**Size of municipality**				
Rural area	73.5 (14,519)	9.1 (1,561)	8.9 (1,496)	8.6 (1,408)
>=100,000 inhabitants	72.6 (18,826)	9.3 (1,952)	9.7 (1,983)	8.4 (1,627)
≥100,000 inhabitants	71.7 (17,657)	10.0 (1,954)	10.1 (1,812)	8.1 (1,405)
Paris	71.7 (9,214)	11.3 (1,016)	10.6 (841)	6.4 (560)
Missing	2,202	263	251	231
**Priority neighborhood**				
No	73.4 (60,789)	9.5 (6,358)	9.2 (5,965)	7.8 (4,956)
Yes	55.4 (1,629)	14.2 (388)	18.2 (418)	12.1 (275)
**Trust in the government**				
Complete trust	85.0 (10,479)	8.3 (719)	4.8 (341)	1.9 (123)
Fair amount of trust	80.5 (30,767)	9.5 (2,903)	7.2 (2,002)	2.8 (760)
Little trust	65.2 (14,388)	11.3 (2,035)	14.2 (2,489)	9.3 (1,681)
No trust at all	51.1 (6,507)	10.1 (1,064)	13.8 (1,486)	25.0 (2,636)
**Trust in scientists**				
Complete trust	83.5 (26,227)	9.0 (2,153)	5.1 (1,058)	2.3 (479)
Fair amount of trust	70.8 (32,717)	10.5 (3,942)	11.3 (4,043)	7.3 (2,608)
Little trust	45.2 (2,507)	9.7 (467)	19.3 (914)	25.8 (1,225)
No trust at all	34.3 (795)	7.9 (161)	15.9 (319)	41.9 (879)

*EpiCov study 3rd wave, July 2021*.

*Data are presented as % (n), except for missing values where only numbers are reported. Among men, 71.1% had had at least one dose at the time of the survey*.

The vaccination rate increased very steadily with age, rising from 54.7% among 18–24-year-olds to 93.2% among 75–84-year-olds, and then falling to 88.2% among those over 85. There were also marked differences in vaccination practices according to social positions. Only 69.8% of people without educational qualifications were vaccinated, compared to 79.2% of those with the highest qualifications. As for the rate of vaccination according to income, it increased regularly from 54.8% among the poorest 10% to 87.6% among the richest 10%. Compared to the mainstream population (74.5%), vaccination uptake was lower only among people belonging to racially minoritized groups, i.e., among first (59.1%) and second-generation immigrants (52.5%) and among people born or whose parents were born in French Overseas Departments (56.2%). Living in a populated area was not associated with being vaccinated, although living in a “priority neighborhood” was (55.4 vs. 73.4%).

Social differences were also found among the unvaccinated: the 10% richest and those with the highest qualifications were more likely to intend to accept vaccination (5.6 and 8.8% respectively) whereas the 10% poorest and people without qualifications were more likely to hesitate (16.9 and 10.9% respectively). Interestingly, racially minoritized first-generation immigrants were among those who least often refused vaccination (7.4%) whereas people from the overseas territories were the most reluctant (14.2%).

**Figure 1:**
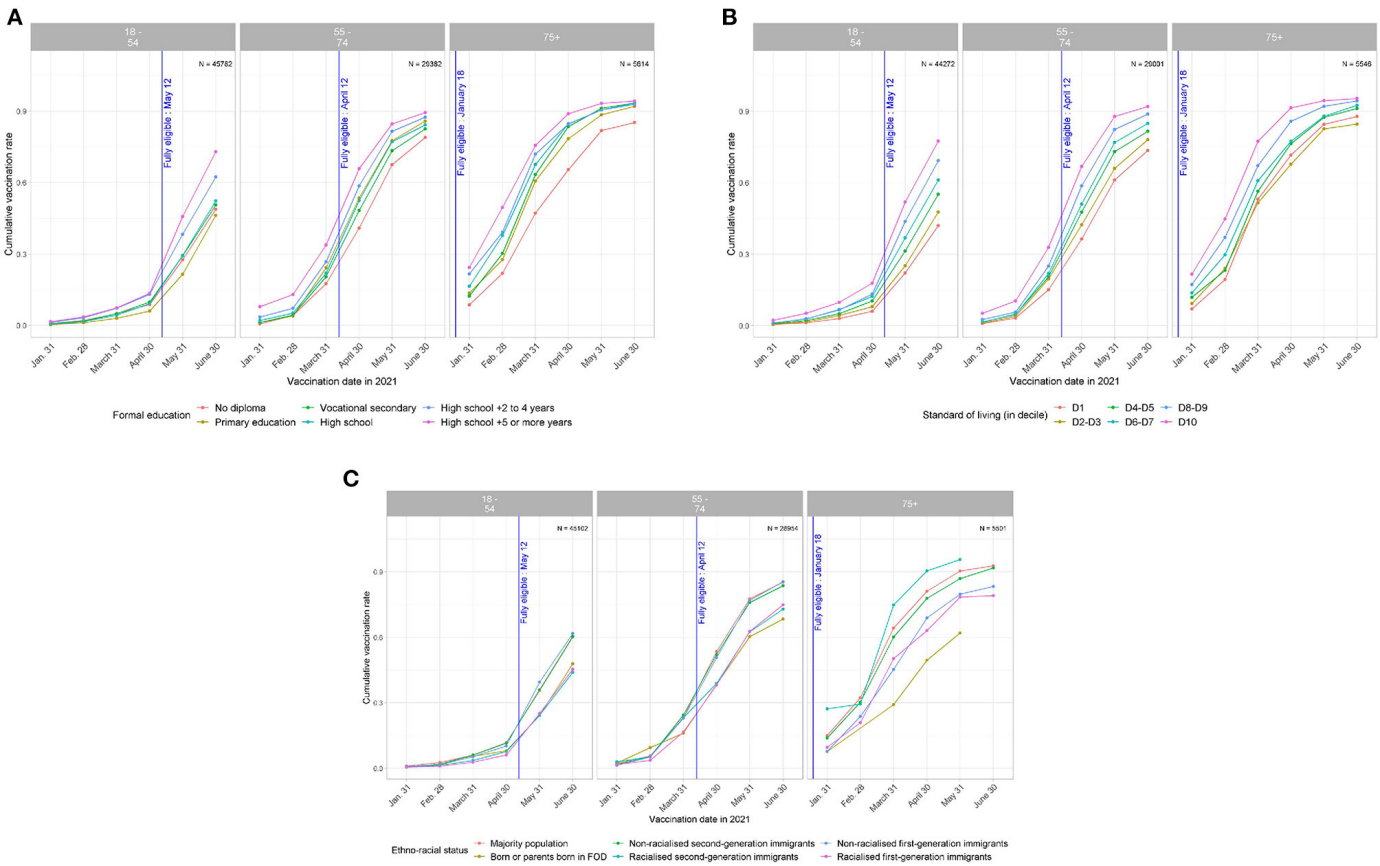
**(A)** Trends over time in vaccination cumulative incidence rates by age, according to level of education. EpiCov study, 3rd wave, July 2021. **(B)** Trends over time in vaccination cumulative incidence rates by age, according to standard of living (in decile). EpiCov study, 3rd wave, July 2021. **(C)** Trends over time in vaccination cumulative incidence rates by age, according to ethno-racial status. EpiCov study, 3rd wave, July 2021.

The data also showed that social differences were present even before vaccination and that they were maintained or widened over time, especially among the 18–54-year-olds (Figures 1A–C). Among these, the gap between the 10% poorest and the 10% richest was 11.9% at the end of April 2021 and it increased to 35.4% by the end of June.

The lack of trust in the government to manage the epidemic crisis was much more pronounced than the lack of trust in the scientists: overall, 15.7% of the respondents trusted the government completely and 17.3% did not trust them at all, while 36.8% of the respondents trusted scientists completely and only 3.9% did not trust them at all (Supplementary Table 1). People at the bottom of the social hierarchy showed much less trust in the government. The differences were similar although less pronounced for trust in scientists. Vaccination status varied greatly and regularly according to the degree of trust in the government: from 85.0% of those who trusted the government completely to manage the epidemic were vaccinated to 51.1% of those who did not trust the government at all (Table 1).

Multivariate analyses confirmed that those not vaccinated were younger, less educated, had lower incomes and more often belonged to racially minoritized groups than vaccinated people in all three sub-groups, especially those who refused to be vaccinated. Multivariate analyses also showed that people's lack of trust in the government and scientists were the factors that were the most strongly associated with refusing to be vaccinated, compared to vaccinated people with an OR of 8.86 (95%CI: 7.13 to 11.01) for complete lack of trust in the government and an OR of 9.07 (7.71 to 10.7) for complete lack of trust in scientists (Table 2).

**Table 2:** Factors associated with vaccination status (multinomial regression, reference = being vaccinated).

	**Intends to get vaccinated**		**Does not know yet**		**Refuses to get vaccinated**	
	**OR**	**95% CI**	**OR**	**95% CI**	**OR**	**95% CI**
**Sex**						
Men	—	—	—	—	—	—
Women	0.85	0.80, 0.90	1.02	0.96, 1.09	1.19	1.10, 1.28
**Age**						
18–24	—	—	—	—	—	—
25–34	1.20	1.06, 1.36	1.34	1.18, 1.52	1.39	1.20, 1.60
35–44	0.83	0.73, 0.93	0.99	0.87, 1.11	0.90	0.78, 1.04
45–54	0.54	0.48, 0.61	0.62	0.55, 0.71	0.58	0.50, 0.67
55–64	0.33	0.29, 0.38	0.47	0.40, 0.54	0.43	0.37, 0.51
65–74	0.20	0.17, 0.24	0.26	0.22, 0.31	0.24	0.20, 0.29
75–84	0.10	0.07, 0.13	0.19	0.15, 0.25	0.20	0.14, 0.27
85+	0.16	0.10, 0.25	0.20	0.13, 0.33	0.47	0.31, 0.72
**Formal education**						
High school +5 or more years	—	—	—	—	—	—
High school +2 to 4 years	1.08	0.97, 1.19	1.20	1.08, 1.33	1.15	1.01, 1.30
High school	1.31	1.17, 1.46	1.27	1.13, 1.44	1.39	1.21, 1.61
Vocational secondary	1.32	1.16, 1.50	1.39	1.21, 1.58	1.32	1.13, 1.54
Primary education	1.29	1.10, 1.50	1.36	1.15, 1.59	1.25	1.03, 1.51
No diploma	1.32	1.11, 1.58	1.59	1.34, 1.89	1.39	1.13, 1.71
**Standard of living (in deciles)**						
D1	—	—	—	—	—	—
D2–D3	1.11	1.00, 1.24	1.40	1.24, 1.58	1.28	1.11, 1.48
D4–D5	1.26	1.12, 1.40	1.51	1.33, 1.71	1.66	1.43, 1.92
D6–D7	1.53	1.36, 1.71	1.76	1.55, 2.00	1.69	1.45, 1.97
D8–D9	1.70	1.51, 1.93	2.13	1.86, 2.43	2.12	1.81, 2.48
D10	1.96	1.71, 2.25	2.37	2.05, 2.74	2.47	2.08, 2.93
**Ethno-racial status**						
Mainstream population	—	—	—	—	—	—
Born or parents born in FOD	1.46	1.16, 1.85	1.55	1.23, 1.96	1.49	1.13, 1.96
Non-racially minoritized second-generation immigrants	1.11	0.98, 1.26	1.14	1.00, 1.30	1.07	0.92, 1.25
Racially minoritized second-generation immigrants	1.31	1.14, 1.51	1.95	1.71, 2.23	1.57	1.33, 1.87
Non-racially minoritized first-generation immigrants	0.94	0.78, 1.13	0.94	0.77, 1.14	1.21	0.98, 1.50
Racially minoritized first-generation immigrants	1.70	1.47, 1.96	1.93	1.65, 2.24	1.58	1.29, 1.94
**Trust in the government**						
Complete trust	—	—	—	—	—	—
Fair amount of trust	1.18	1.06, 1.31	1.21	1.05, 1.40	1.00	0.80, 1.25
Little trust	1.57	1.40, 1.75	2.48	2.15, 2.87	3.17	2.55, 3.94
Not trust at all	1.79	1.58, 2.03	3.12	2.68, 3.63	8.86	7.13, 11.0
**Trust in scientists**						
Complete trust	—	—	—	—	—	—
Fair amount of trust	1.17	1.09, 1.25	2.25	2.07, 2.45	2.87	2.55, 3.22
Little trust	1.34	1.18, 1.52	4.08	3.63, 4.58	8.62	7.53, 9.87
Not trust at all	1.22	0.99, 1.51	3.19	2.67, 3.80	9.07	7.71, 10.7

*EpiCov study 3rd wave, July 2021*.

*Also adjusted for social class, healthcare worker, cohabitation status, has children, population size of municipality, priority neighborhood, perceived health status, COVID-19 comorbidities, knows someone who has had a severe form of COVID-19, COVID-19 risk perception, positive test in last 6 months, and date of response to questionnaire*.

**Figure 2:**
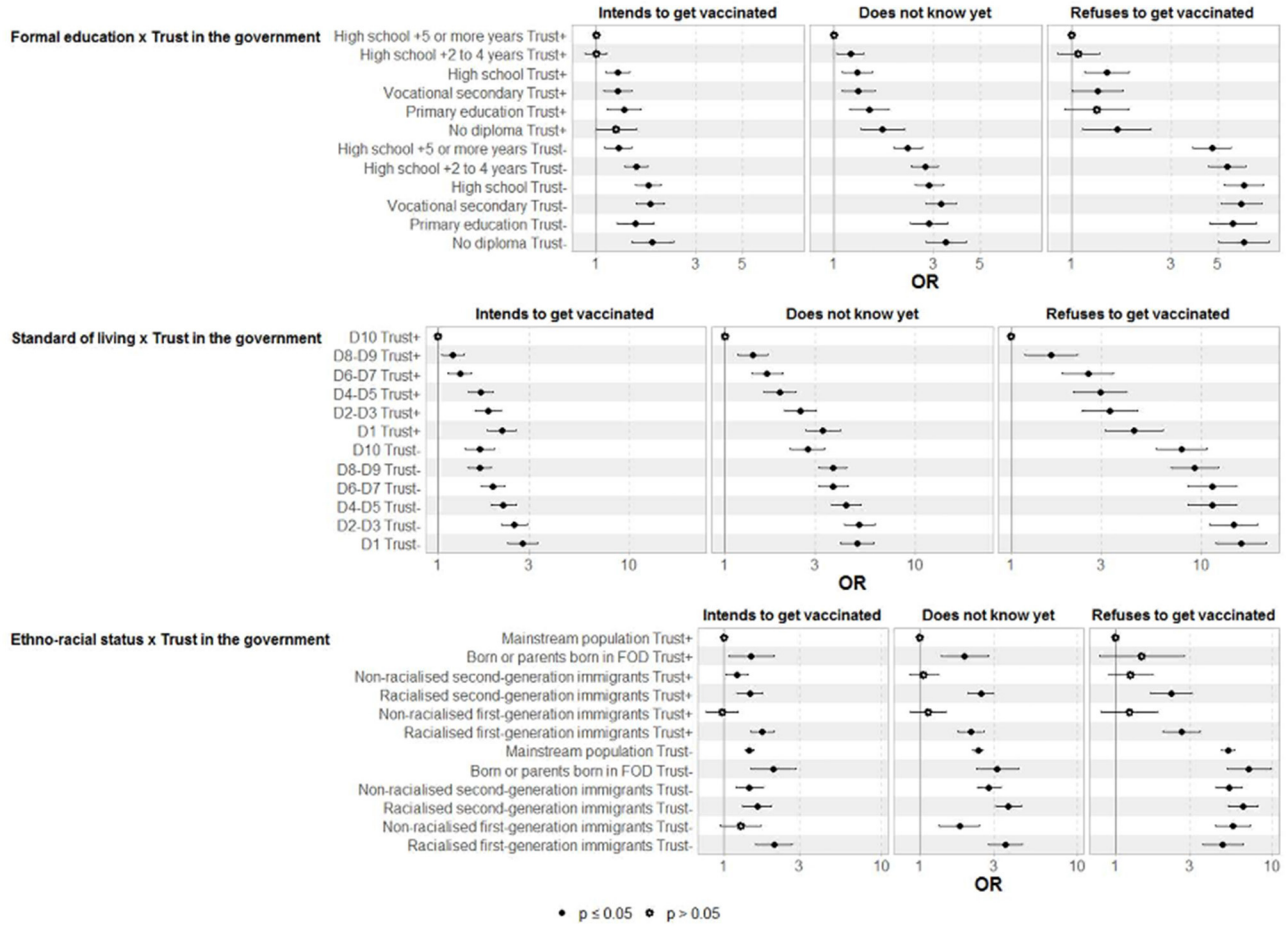
Interaction between trust in the government (yes or no) and (i) diploma, (ii) standard of living and (iii) ethno racial status and vaccination status. Multinomial regression (ref = being vaccinated). EpiCov study, 3rd wave, July 2021.

The data also showed that the richer people were, the stronger the effects of trust in the government were on the decision not to refuse to get vaccinated (Figure 2; Supplementary Table 2A). Compared to the richest 10% who trust the government, the poorest 10% who also did reached an OR of 4.44 (3.13 to 6.31) for the decision to refuse to get vaccinated, the poorest 10% who did not trust the government reached an OR of 16.2 (11.9 to 22.0) (Figure 2; Supplementary Table 2A). Similar but less marked differences were found according to formal education. Finally, the effect of trust in the government on decreasing refusal to get vaccinated was less pronounced among the racially minoritized first and second generations compared to the mainstream population.

The results were similar but to a lesser extent for mistrust in scientists (Supplementary Table 2B).

## Discussion

EpiCov is among the largest national socio-epidemiological cohort surveys to be conducted on a random sample of the population, simultaneously considering gender, class and ethno-racial status, health data, and trust of the government and of scientists to analyse social inequalities in vaccination.

We found marked social and ethno-racial inequalities in vaccination, in a context of free access to vaccination and at a time when anti-COVID certifications had not yet been made restrictive. The least educated, those with the lowest incomes, and racially minoritized groups were less likely to have been vaccinated and these differences were maintained or increased over time. People's lack of trust in the government and scientists to manage the health crisis remained the factor that was the most strongly associated with refusing to get vaccinated. The impact of trust on not refusing to be vaccinated was even more marked among people at the top of the social hierarchy, thus reinforcing social inequalities in vaccination.

With regard to the social barriers to access to vaccination, we should first of all note that the lower vaccination rates among younger people are likely to be related to a shorter access period. Indeed, data was collected in July 2021, at a time when COVID-19 vaccines were available in France for any individuals aged 18 and over as of May 12th 2021 (12th April for people over 55, and 18th January 2021 for people over 75). Secondly, it is interesting to note that social differences in vaccination practices overlapped with the social distribution of vaccine reluctance observed in France 8 months earlier, except for gender differences (16). Indeed, women were no less likely to be vaccinated than men, although they were more reluctant to get vaccinated in France, as in many countries, before the vaccine was made available to all (30). Faced with the reality/possibility of prevention, one could hypothesize that their gendered reflexes as guardians of the family's health came into play (31). Our results also showed that those with lower levels of education and those belonging to the working class were less likely to be vaccinated, as found in the UK (7). Although many epidemiological studies have shown that the less educated were more reluctant to be vaccinated, they do not explore sociological hypotheses to account for these statistical correlations (32). One could wonder if this does not translate the fact that members of the working classes have a perception of their body and their health that is more distant from medical diagnoses and recommendations than the upper class (33). Racially minoritized groups also appeared to be less likely to be vaccinated, as found in British and US surveys (5, 8, 34, 35). Numerous studies have shown that racially minoritized groups (29, 36) have less confidence in the healthcare system and in caregivers than the mainstream population (37, 38). This lack of trust particularly results from discrimination and mistreatment to which these populations have been exposed when resorting to the public health system (39, 40). A recent study among students in London showed that experiences of racial discrimination increased the likelihood of subsequent COVID-19 vaccine refusal nearly four-fold (5). Barriers other than experiences of discrimination should also be considered, such as the lack of health insurance coverage in countries where vaccination is not free (41). In this respect, it is surprising to note that significant differences were recorded according to income level in the multivariate model, despite vaccination being free in France. While the poorest have the same tendency as others to comply with the use of masks in France (42), they are less likely to be vaccinated. The exclusion of the poorest part of the population from the social contract could lead to a diminished sensitivity toward the national solidarity dimension of vaccination, strongly emphasized in the public discourse on prevention in France. The low rates of vaccination among the most deprived, also found in a US survey (6), probably also relates to the fact that they generally have poor access to healthcare than others for given needs (10).

Our results underline the need to develop outreach strategies targeting the poorest, the least educated, and racially minoritized groups, as recommended by Hanif back in 2020 (43). However, given the preponderant place of vaccine refusal due to lack of trust in the government's and scientists' attempts to curb the spread of the coronavirus, the characteristics of the messenger in vaccination campaigns should also be considered. A recent study compared the relationship between government trust and vaccination coverage in 177 countries, but using pre-pandemic trust scores (44). Studies in the US have shown that non-uptake of vaccination is higher in counties where conservative votes are higher (15, 45). However, in a context where the abstention rates are high (46), especially in France, it seems more relevant to consider the link between trust in the government and individual decision-making about vaccination. We found that lack of trust in the government and in scientists to curb the spread of the epidemic was the strongest predictor for not being vaccinated, even after adjustment on social factors, which were shown to be low confounding factors in a supplementary analysis (data not shown). Nevertheless, the effects of trust were less pronounced for people at the lower end of the social ladder and for racially minoritized groups, with the reinforcement of social inequalities in vaccination as a consequence. It thus seems preferable for the preventive discourse to come from health agencies in close collaboration with community organizations and social workers (47), without political interference. People's lack of trust in scientists could reflect a strong connivance, in France, between the government and the scientific council. It could also reflect doubts arising from the contradictory injunctions that have been made in the media. Finally, suspicions of scientists colluding with big pharmaceutical companies could also contribute to explaining this lack of trust (48).

It should also be emphasized that the spread of new variants, which has led to a further outbreak of the epidemic in France and in many countries with high vaccination rates, raises questions for many people about the effectiveness of vaccination. New strains, the requirement for boosters, the uncertainty of a possible herd immunity, and the complexity of the scientific and political discourse on COVID-19 vaccines could prompt concerns that groups of people in the population who are more distant from health literacy may no longer embrace the COVID-19 vaccine.

Our analysis nevertheless has some limitations. First, as any national population-based survey, the present study failed to capture highly vulnerable groups such as undocumented migrants and homeless people, who were particularly affected by the pandemic (49).

Secondly, our analysis was based on a survey conducted in July 2021. Until reaching a plateau in October 2021 (50), vaccination rates continued to rise particularly in connection with the mandatory anti-COVID-19 certification introduced on July 21st 2021 (3, 51). Considering that the least privileged social groups are less impacted by the anti-COVID-19 certification, since they are not likely to routinely access places like restaurants, we could hypothesize that the social inequalities observed are still present today, even if their magnitude is less prominent. In addition, it was interesting to study the social inequalities in vaccination practices before the introduction of the mandatory anti-COVID-19 certification to be able to evaluate its effectiveness afterwards.

The highly structuring effect of trust in the government and scientists remains to be understood in greater detail. The role of the social networks and the contradictory information on COVID-19 vaccination (34) is particularly difficult to grasp in a quantitative survey.

Finally, the issue of social inequalities in vaccination practices is all the more important because the social groups that are the least vaccinated are also those most at risk of contracting COVID-19 (1). Our analyses show that a top-down conception of preventive policies comes up against the social logics that structure vaccination status. There is an urgent need to depoliticise vaccination strategies, and to develop outreach programmes for the most socially disadvantaged groups but also “culturally competent” vaccination campaigns (43) conceived with people from different social and racial backgrounds to enable them to make fully informed choices.

## Summary Boxes

**What is already known on this topic**

Some studies in the UK and in the US have shown that the most socially disadvantaged and racially minoritized groups are the least vaccinated, and that trust in the government was strongly associated with vaccine hesitancy.

**What this study adds**

We found social and ethnoracial inequalities in vaccination practices, which result from social barriers to engaging in prevention practices. But above all, people's lack of trust in the government and scientists was the factor most strongly associated with refusing to get vaccinated. Nevertheless, the effects of trust on not refusing to get vaccinated were less pronounced for people at the lower end of the social ladder and for those who belong to racially minoritized groups, leading to the reinforcement of social inequalities in vaccination.

**How this study might affect research, practice or policy**

Our results show the need to develop outreach strategies with no interference of politics, delegated to key-players able to design targeted preventive messages conceived with people from different social and racial backgrounds to enable people to make fully informed choices.

## Data Availability Statement

The data analyzed in this study is subject to the following licenses/restrictions: Data of the study are protected under the protection of health data regulation set by the French National Commission on Informatics and Liberty (Commission Nationale de l'Informatique et des Libertés, CNIL) in line with the European regulations and the Data Protection Act. The data can be available upon reasonable request to the co-principal investigator of the study (nathalie.bajos@inserm.fr). The French law forbids us to provide free access to EPICOV data; access could however be given by the EPICOV steering committee after legal verification of the use of the data. Please, feel free to come back to us should you have any additional questions.

## Ethics Statement

The survey was approved by the CNIL (French Independent Administrative Authority responsible for data protection) on 25 April 2020 (ref: MLD/MFI/AR205138) and by the ‘Comité de protection des personnes' (French equivalent of the Research Ethics Committee) on 24 April. Written informed consent to participate in this study was provided by the participants' legal guardian/next of kin.

## EpiCov Study Group

Nathalie Bajos (co-principal investigator), Josiane Warszawski (co-principal investigator), Guillaume Bagein, Emilie Counil, Florence Jusot, Nathalie Lydie, Claude Martin, Laurence Meyer, Philippe Raynaud, Alexandra Rouquette, Ariane Pailhé, Delphine Rahib, Patrick Sillard, Rémy Slama, Alexis Spire.

## Author Contributions

NB and ASp had full access to all the data in the study and took responsibility for the integrity of the data and the accuracy of the data analysis. Study concept and design: NB and ASp. Data acquisition: IPSOS. Data analysis and interpretation: NB, ASp, J-EF, and LS. Drafting of the manuscript: NB, ASp, J-EF, and LS. Critical revision of the manuscript for important intellectual content: all authors. Statistical analysis: ASi. Study supervision: NB and ASp. All authors contributed to the article and approved the submitted version.

## Funding

This work was supported by Inserm (Institut National de la Santé et de la Recherche Médicale), the French Ministry for Research, and the DREES (Direction de la recherche, des études, de l'évaluation et des statistiques). The funders facilitated data acquisition but had no role in the design, analysis, interpretation, or writing. This project has received funding from the European Union's Horizon 2020 research and innovation programme under grant agreement No. (101016167), ORCHESTRA (Connecting European Cohorts to Increase Common and Effective Response to SARS-CoV-2 Pandemic). NB has received funding from the European Research Council (ERC) under the European Union's Horizon 2020 research and innovation programme (grant agreement No. [856478]), and from Horizon 2020 European research Council (Gendhi-Synergy grant agreement No. [SGY2019-856478]). The funders had no role in study design, data collection and analysis, decision to publish, or preparation of the manuscript.

## Conflict of Interest

The authors declare that the research was conducted in the absence of any commercial or financial relationships that could be construed as a potential conflict of interest.

## Publisher's Note

All claims expressed in this article are solely those of the authors and do not necessarily represent those of their affiliated organizations, or those of the publisher, the editors and the reviewers. Any product that may be evaluated in this article, or claim that may be made by its manufacturer, is not guaranteed or endorsed by the publisher.
